# Evaluation of Plasma Neurofilament Light Chain Levels as a Biomarker of Neuronal Injury in the Active and Chronic Phases of Autoimmune Neurologic Disorders

**DOI:** 10.3389/fneur.2022.689975

**Published:** 2022-03-02

**Authors:** Ryan Kammeyer, Christopher Mizenko, Stefan Sillau, Alanna Richie, Gregory Owens, Kavita V. Nair, Enrique Alvarez, Timothy L. Vollmer, Jeffrey L. Bennett, Amanda L. Piquet

**Affiliations:** ^1^Department of Neurology, University of Colorado Anschutz Medical Campus, Aurora, CO, United States; ^2^Department of Pharmacy, University of Colorado Anschutz Medical Campus, Aurora, CO, United States; ^3^Department of Ophthalmology, University of Colorado Anschutz Medical Campus, Aurora, CO, United States

**Keywords:** autoimmune encephalitis (AE), neurofilament (NF), biomarker, autoimmune neurological disorders, neurofilament light (NfL) chain, cerebellar ataxia

## Abstract

**Objective:**

To evaluate plasma neurofilament light (NfL) levels in autoimmune neurologic disorders (AINDs) and autoimmune encephalitis (AE).

**Background:**

Each particular neural autoantibody syndrome has a different clinical phenotype, making one unifying clinical outcome measure difficult to assess. While this is a heterogeneous group of disorders, the final common pathway is likely CNS damage and inflammation. Defining a biomarker of CNS injury that is easily obtainable through a blood sample and reflects a positive treatment response would be highly advantageous in future therapeutic trials. Measurement of blood concentration of neurofilament light (NfL) chain, however, may provide a biomarker of central nervous system (CNS) injury in AE and other AINDs. Here we provide an initial evaluation of plasma NfL levels in AE as well as other AINDs during active and chronic phases of disease and demonstrate its potential utility as a minimally-invasive biomarker for AE and AINDs.

**Design/Methods:**

Patients were retrospectively identified who were enrolled in the biorepository at the Rocky Mountain MS Center at the University of Colorado, or were prospectively enrolled after initial presentation. Patients had a well-defined AIND and were followed between 2014 and 2021. NfL was tested using the Single Molecule Array (SIMOA) technology. Patients with headaches but without other significant neurologic disease were included as controls.

**Results:**

Twenty-six plasma and 14 CSF samples of patients with AINDs, and 20 plasma control samples stored in the biorepository were evaluated. A positive correlation was found between plasma and CSF NfL levels for patients with an AIND (*R*^2^ = 0.83, *p* < 0.001). Elevated plasma levels of NfL were seen in patients with active AE compared to controls [geometric mean (GM) 51.4 vs. 6.4 pg/ml, *p* = 0.002]. Patients with chronic symptoms (>6 months since new or worsening symptoms) of AE or cerebellar ataxia (CA) showed a trend toward lower plasma NfL levels (GM 15.1 pg/ml) compared to active AE or CA. Six patients with longitudinal, prospective sampling available demonstrated a trend in decreased plasma NfL levels over time.

**Conclusions:**

Our findings support the use of plasma NfL as a potential minimally-invasive biomarker of CNS injury.

## Introduction

Over the past decade, multiple autoimmune neurologic disorders (AINDs) mediated by pathogenic neuronal cell surface antibodies (neuronal surface antibody syndromes, or NSAS) have been identified. AINDs encompass all neurologic isolated inflammatory disease thought to be mediated by the adaptive immune system–including autoimmune encephalitis (AE), stiff person spectrum disorder (SPSD), autoimmune cerebellar syndromes, and demyelinating diseases like neuromyelitis optica spectrum disorder (NMOSD) or anti-myelin oligodendrocyte (MOG) antibody disease (MOGAD). Both observational and retrospective studies have reported improved clinical outcomes with immunotherapy (1–5) in AINDs; however, there remains a strong need for randomized, controlled clinical trials to establish a standard of care for the treatment of AE and the non-demyelinating AINDs. While change in seizure frequency and cognitive functional status have been used as outcome measures for AE therapy, these measures are problematic end-points due to their poor specificity and sensitivity across the heterogenous presentations of even AEs caused by the same autoantibody (6). Some AEs may cause neuronal destruction, while others may cause dysfunction only by blocking signaling, interfering with synaptic architecture, or receptor internalization. Therefore, defining a unifying, quantitative biomarker of central nervous system (CNS) injury in AEs that is readily obtainable through a blood sample would significantly advance clinical research.

Neurofilaments are neuron-specific cytoskeletal proteins that are released following axonal damage (7). Elevated levels of NfL have been interpreted as reflecting axonal damage and neuronal death in MS (7, 8), neurodegenerative dementia (9–11), and motor neuron disease (12, 13). In MS, NfL in serum highly correlates with CSF levels (14). In addition to correlating with disease activity on MRI (7), NfL serves as a promising prognostic and therapeutic biomarker in MS (14–16).

Prior studies have described CSF levels of NfL in AE (17–19), however there is little data on serum NfL levels in AE (20). One study examined CSF levels of NfL in a cohort of 25 subjects with autoimmune encephalitis (including seronegative antibody syndromes, NSAS [*n* =5; 4 NMDAR and 1 LGI1], and intracellular antibody syndromes) with evidence of elevated CSF NfL at the time of diagnosis correlating to disability at 1 year (18). An additional retrospective study examined progranulin (PGRN) in both serum and CSF in 38 patients with AE [NMDAR *n* = 18, Caspr2 *n* = 8, LGI1 *n* = 10, GABA-bR *n* = 1, and AMPAR *n* = 1]; CSF NfL (*n* = 25) and t-tau (*n* = 13) was also measured in these patients (17). In this cohort, 3 NMDAR patients had highly pathological CSF NfL levels that seemed to best characterize the state of neuronal death in the brain. These studies had evaluated CSF NfL using commercial enzyme-linked immunosorbent assay (ELISA; UmanDiagnostics AB, Umeå, Sweden). Another recent study of 25 patients with autoimmune encephalitis (NMDAR, *n* = 10; LGI-1, *n* = 9; Caspr2, *n* = 3; both LGI1 and Caspr2, *n* = 1, GABA-bR, *n* = 1; AMPAR, *n* = 1), demonstrated elevated serum levels of NfL that correlated with elevated levels of CSF NfL (20). This particular study, similar to our study, used a highly sensitive assay for NfL testing using the SIMOA platform.

In our study, we evaluated NfL in the plasma of 26 patients with various AINDs, along with 20 control patients looking at both active and chronic phases of each AIND. Fourteen of 26 AIND patients also had matched CSF available for NfL testing.

## Materials and Methods

### Patients

Patients enrolled in the biorepository specimen bank at the University of Colorado Anschutz Medical Campus from 2014 to 2021 were identified retrospectively. Between 2019 and 2021, patients who were evaluated in the Autoimmune and Neuroimmunology/Multiple Sclerosis outpatient clinics or inpatient neurology service at the University of Colorado with a well-defined AIND were enrolled prospectively into our autoimmune, paraneoplastic and inflammatory neurological disease registry and biorepository. A fellowship-trained Neuroimmunologist made diagnosis of a well-defined AIND. Patients who had been enrolled in the biorepository specimen bank for a primary evaluation of headache without other significant neurologic symptoms were identified retrospectively to serve as a control group. All patients or legal representatives consented to enrollment in the autoimmune, paraneoplastic and inflammatory neurological disease registry and biorepository [approved by the Colorado Multiple Institutional Review Board (COMIRB)].

We included 26 patients with CNS autoimmune neurological syndromes, with 18 of these patients having active symptoms, and 8 having chronic symptoms. Patients were defined as having active AE if they had experienced new or worsening symptoms of altered mental status, impaired cognition/memory, personality/behavioral change, seizure frequency, decreased speech or mutism, or centrally-mediated movement disorders (ataxia, chorea) in the past 6 months, while being on or off immunotherapy. Patients were defined as having active SPSD if they had experienced new or worsening symptoms of SPSD (muscle rigidity/spasms, hyperstartle) without concurrent encephalitic symptoms in the past 6 months. Patients were defined as having active autoimmune cerebellar ataxia (active CA) if they had experienced worsening of cerebellar symptoms without concurrent encephalitic symptoms in the past 6 months. Active symptoms for all patients could be either at initial disease onset or during a relapse. Patients who had experienced no recent new or worsening encephalitic or cerebellar symptoms in the 6 months prior to sample collection were defined as having chronic autoimmune encephalitis /cerebellar ataxia (chronic AE/CA). All patients were included only once in analysis based on clinical presentation at the time of initial sampling. A cut-off of 6 months for active (recent) symptoms was chosen based on data regarding serum NfL levels in stroke, as this is a monophasic neurologic injury, showing return to levels of healthy controls at around 6 months post-injury (21). An additional 20 patients with a primary headache disorder such as migraine (excluding patients with idiopathic intracranial hypertension) were included as non-inflammatory neurologic controls.

Demographics of each patient and associated autoantibodies, neurologic symptoms, and results of diagnostic testing at the time of initial presentation were obtained through retrospective chart review.

### Autoantibody Detection

The presence of serum and CSF autoantibodies to neuronal autoantigens included NMDAR, LGI1, DPPX, GAD65, GlyR, GFAP, TRIM46, and GABA-aR. All antibodies were tested at Mayo Clinic Laboratories with the exception of GABA-aR antibody testing, which was tested at Hospital Clinic, University of Barcelona, Spain. GlyR, GFAP, and TRIM46 antibody testing was performed on cell-based assay (CBA) on a research basis, while all other antibody testing at Mayo clinic was performed on a commercially available basis at that time; all neuronal cell surface antigens (NMDAR, LGI1, DPPX) were performed using CBA.

### NfL Analysis

NfL was tested in retrospective and prospective samples, including 26 plasma and 14 CSF samples of patients with AINDs, and 20 control patients. CSF was collected, centrifuged immediately to remove cells and stored at −80°C until analysis. Plasma was obtained in sodium citrate tubes then aliquoted at room temperature and stored at −80°C. Measurement of NfL concentration was performed in duplicates for all samples using the SIMOA Nf-light kit^®^ (Quanterix SR-X™ by Simoa^®^ platform).

### Theory/Calculation

**Figure 1:**
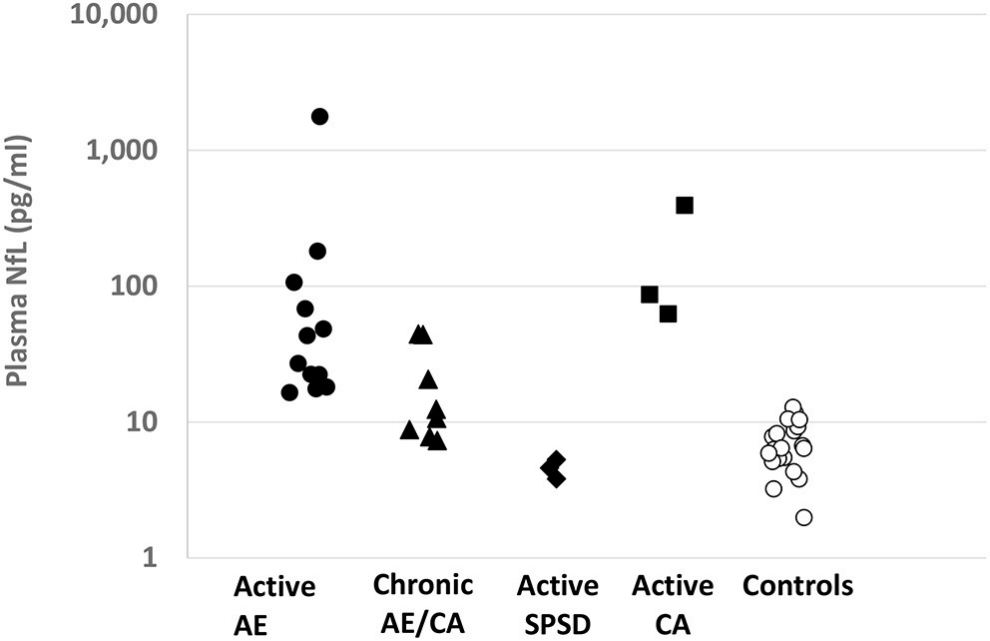
Plasma NfL levels by Autoimmune Neurological Disease Category: Plasma NfL levels (pg/ml) are provided for each defined patient group on a logarithmic scale. AE, autoimmune encephalitis; CA, cerebellar ataxia; SPSD, stiff person spectrum disorder.

**Figure 2:**
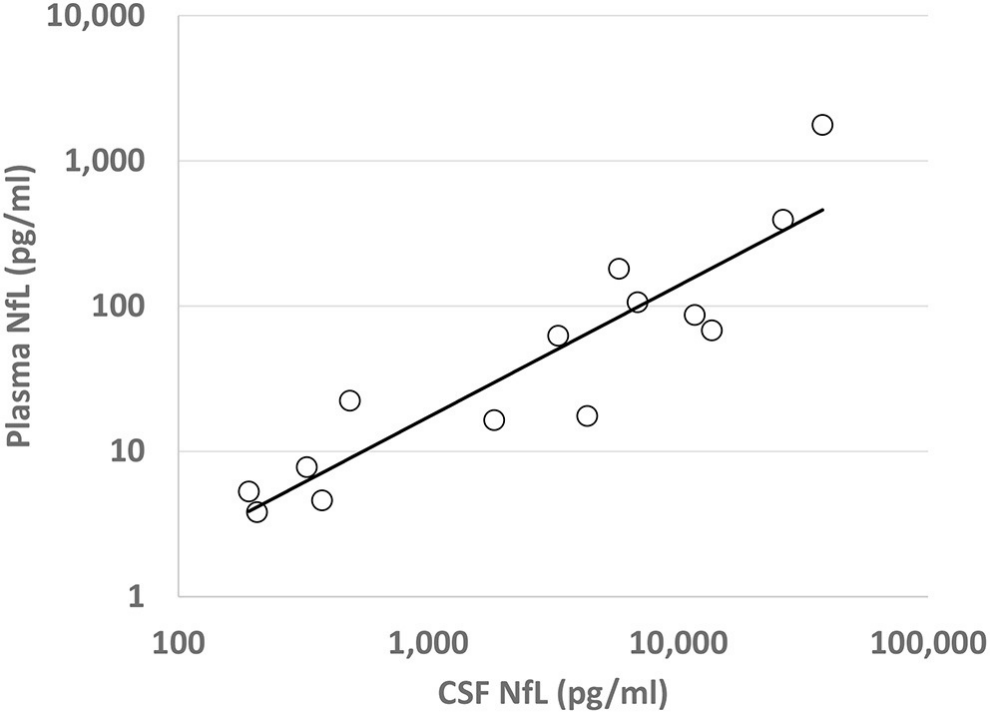
Plasma and Cerebrospinal Fluid NfL Correlation: Correlation of transformed (log_10_) plasma and CSF NfL levels (pg/ml) for each patient with an AIND, with regression line of best fit shown. *R*^2^ = 0.83, *p* < 0.0001.

Plasma and CSF NfL were logarithmically transformed to reduce skew. By group, summary statistics are presented for plasma NfL (Figure 1) and differences in mean among groups were analyzed with an ANOVA type model. Different groups were permitted to have different residual variances, and denominator degrees of freedom were determined by the Satterthwaite method. Omnibus F tests tested whether there were any mean differences among all groups and the non-control groups. Pair-wise comparisons were performed with *T*-tests, with the Tukey-Kramer adjustment considered to control the family-wise error rate for all pair-wise comparisons. For a subset of non-control patients where CSF NfL was available, Pearson correlations were run for plasma and CSF NfL (Figure 2). Analysis was performed in SAS 9.4, STATA 15.1, and R 3.6.1.

## Results

Patient demographics and clinical presentations are summarized in Table 1. Patients with either active AE or CA tended to be older [median 64 years-old (yo) and 75 yo], compared to those with active SPSD (median 49 yo) and controls (median 48 yo). Patients with chronic AE/CA were also older (median 64 yo) compared to the active SPSD and control groups. However, age demographics between active AE and chronic AE/CA were similar. Diagnostic testing (MRI, EEG, and/or CSF) were abnormal in the majority of patients with encephalitic or cerebellar symptoms. Plasma NfL levels in patients with chronic AE/CA were obtained a median of 10 months after the last episode of symptom worsening (range 7–108 months).

**Table 1:** Demographic and clinical data of patients.

	**Active AE**	**Chronic AE / CA**	**Active SPSD**	**Active CA**	**Controls**
Number of patients	12	8	3	3	20
Age, years, median (range)	64 (23–78)	64 (18–78)	49 (39–55)	75 (63–78)	48 (27–63)
Female, n (%)	6 (50)	6 (75)	1 (33)	3 (100)	14 (70)
**Neurologic symptoms at onset of disease**, ***n*** **(%)**					
Cognitive dysfunction	12 (100)	7 (88)	SPSD: 3 (100)	Ataxia: 3 (100)	Headache: 20 (100)
Psychiatric symptoms	5 (42)	3 (38)			
Seizures	5 (42)	3 (38)			
Ataxia		1 (13)			
Abnormal diagnostic testing^*^, ***n*** **(% of performed tests)**					
MRI	8 (67)	4 (50)	0 (0)	1 (33)	1 (6)
EEG	8 (80)	5 (83)	-	-	-
CSF	10 (91)	3 (43)	1 (33)	3 (100)	3 (16)
**Antibody status**, ***n*** **(%)**					
	NMDAR, 1 (8) LGI1, 2 (17) GAD65, 2 (17) GlyR, 1 (8) GFAP, 1 (8) LE, 2 (17) Ab negative AE, 3 (25)	NMDAR (AE), 1 (13) NMDAR (isolated ataxia), 1 (13) LGI1, 1 (13) DPPX, 1 (13) GABA-aR/GAD65, 1 (13) GAD65, 1 (13) Ab negative AE, 2 (26)	GlyR, 2 (66) LGI1, 1 (33)	GAD65, 1 (33) TRIM46, 1 (33) Paraneoplastic cerebellar degeneration, 1 (33)	N/A
Plasma NfL, pg/ml, geometric mean (range)	51.4 (16.4–1,768)	15.1 (7.3–44.4)	4.5 (3.8–5.3)	128.8 (62.5–393.4)	6.4 (2.0–12.8)
CSF NfL, pg/ml, median (range)	1,161 (486–37,818)	327	207 (192–376)	11,650 (3,311–26,274)	476 (159–3,423)

* *MRI abnormalities included unilateral or bitemporal T2 hyperintense signal, parenchymal or leptomeningeal contrast enhancement, hippocampal atrophy, cerebellar degeneration, and extensive white matter disease. EEG abnormalities included diffuse or focal slowing, epileptiform discharges, or electrographic/electroclinical seizures. CSF abnormalities included >5 nucleated cells /mm^3^, >2 unique CSF oligoclonal bands, or protein > 50 mg/dl*.

Elevated plasma NfL levels were seen in patients with active AE/CA compared to controls [geometric mean (GM) 51.4 vs. 6.4 pg/ml, *p* = 0.002] as shown in Table 1, Figure 1. Elevated plasma NfL was seen regardless of presence of MRI abnormalities. The group of patients with chronic AE/CA showed a non-significant trend toward lower plasma NfL levels (GM 15.1 pg/ml) when compared to active AE or active CA [GM 51.4 pg/ml (*p* = 0.11) and 128.8 pg/ml (*p* = 0.15), respectively]. Notably, the three patients with plasma NfL levels above our control range were 7, 8, and 9 months out from their last new or worsening of symptoms (ages of each patient were 46, 78, and 73 yo, respectively); the remainder were 9 or more months out. Active CA also showed elevated plasma NfL (GM 128.8 pg/ml). Active SPSD showed lower plasma NfL (GM 4.5 pg/ml) compared to both active AE (*p* < 0.001) and chronic AE/CA (*p* = 0.014).

A correlation between CSF NfL and plasma NfL was noted for all AINDs (*R*^2^ = 0.83, *p* < 0.001), as shown in Figure 2. For patients with active AE, no significant correlation was found between the initial plasma NfL and the Modified Rankin Score (mRS) at 1 year after presentation (*R*^2^ = 0.404, *p* = 0.06, *n* = 9), nor was a correlation found for CSF NfL (*R*^2^ = 0.420, *p* = 0.16, *n* = 6).

**Figure 3:**
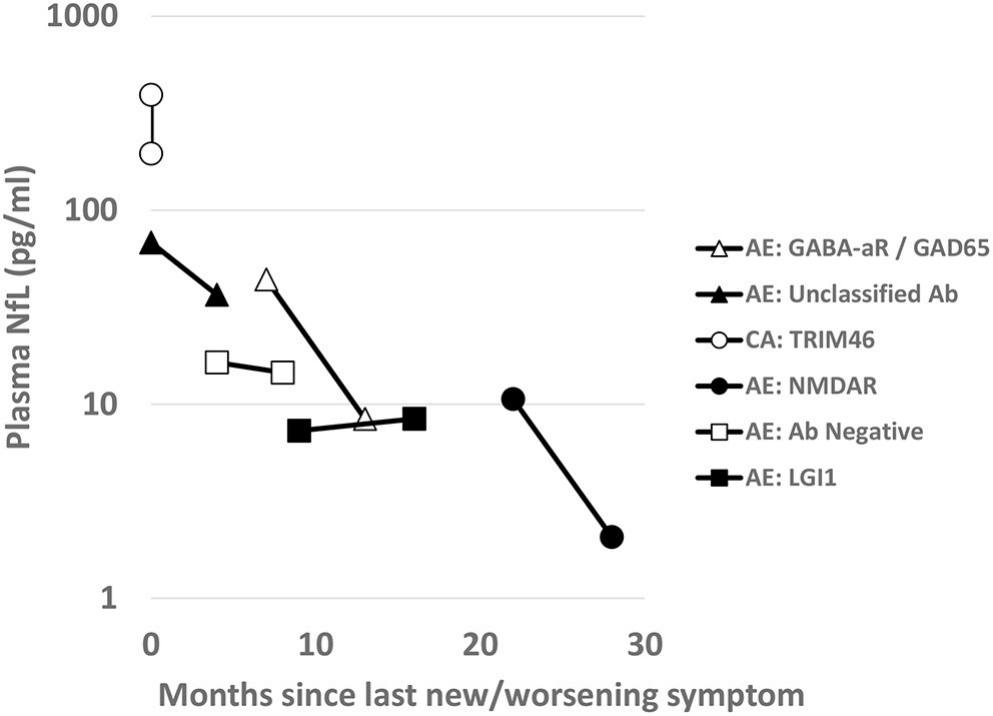
Longitudinal Plasma NfL: For the six AIND patients with longitudinal plasma sample collections, NfL levels (pg/ml) are shown on a logarithmic scale over time. Scale shows collections over months since onset of neurological symptoms or time since last clinical relapse of their disease. Between the first and second time point for each sample patients were treated with variable immunotherapies (TRIM46 with steroids with only 3 weeks in between sampling; unclassified antibody with steroids and rituximab; antibody negative AE with steroids and rituximab; for the remainder [NMDA, LGI, GABA-aR/GAD65], they were on maintained on rituximab therapy.

Six patients had subsequent prospective longitudinal samples obtained. These individual patients and the trend of the plasma NfL levels for each AIND relative are shown in Figure 3. Each patient had immunotherapy initiation close to initial sample collection (range 3 months prior to 1 month after sample collection).

## Discussion

In this study, we used a highly sensitive assay to detect NfL levels in the plasma of patients with AINDs–including active AE, active SPSD, active CA, and chronic AE/CA.

We demonstrated a statistically significant elevation in plasma NfL levels in patients with active AE compared to our controls (*p* = 0.002) and those with SPSD (*p* < 0.001). Similar to other published studies, NfL levels tested at follow up during chronic disease tended to reflect lower NfL levels; however, the majority of these studies focused on CSF NfL (17–19) with one study demonstrating this trend in 13 follow up samples (20).

Looking at the trajectory of plasma NfL for individual patients with AINDs after initial immunotherapy, we can see that each individual demonstrated a different downtrend of plasma NfL after immunotherapy initiation. The differences in trajectory may be due to sampling times (decrease may not be linear), differences in demographics, AIND subtype, or immunotherapy regimen. It is notable that NfL may remain elevated relative to our neurologic control patients for > 1 year after immunotherapy initiation; this has been noted in patients after a monophasic traumatic brain injury as well (22–24). Further studies may look at whether this is a chronic state or if the NfL level would decrease further with additional immunotherapy, and if differences in trajectories for a similar AIND would be seen between different immunotherapy regimens.

We were not able to demonstrate a prognostic value of plasma or CSF NfL in our cohort for active AE–there was no significant correlation between these and the 1-year mRS. This may have occurred for several reasons: (1) a variety of autoantibody and encephalitic syndromes were included in our analysis–it may be that NfL levels vary too widely between these different syndromes, and while it may be prognostic for one syndrome, it may not be prognostic for all. Clinical scores such as the anti-NMDAR encephalitis 1-year functional status (NEOS) score have been developed recently to assist in prediction of 1-year functional status (25)–but even these has only been validated in cohorts of anti-NMDAR encephalitis; (2) It may be that initial NfL levels are prognostic, but our small sample size limited our ability to detect this; or (3) it may be that these levels are not prognostic, with other factors such as duration of symptoms, age, specific autoantibody syndrome, or specific immunotherapy regimens giving greater weight to eventual functional outcome (26).

We did not have any patients with longitudinal sampling have a significant clinical relapse during the study period, so we were not able to assess the predictive values of initial NfL on risk of subsequent relapse. There was not a difference noted between plasma NfL levels in active encephalitic symptoms in initial presentation verses relapse, although longitudinal data prior to relapse in this cohort was lacking. In stable patients on maintenance therapy for AE, having a predictive biomarker for subsequent relapse risk would have great utility. As older age may correlate with both outcome of AE (26) and NfL levels, further longitudinal studies looking at NfL levels, relapse rates, and functional outcomes in a defined age range may be of use in determining the predictive value of NfL.

For the other AINDs investigated in this study, the active autoimmune ataxias showed similar or greater elevations in plasma NfL levels–this is likely due to a greater number of paraneoplastic syndromes within this group. Paraneoplastic cerebellar ataxia syndromes thought to be largely T-cell mediated, may cause greater neuronal destruction. In the cases of SPSD, while the exact pathophysiology remains elusive, it is thought that impairment of the GABA inhibition pathway leading to motor hyperactivity plays a key role in the symptomatology. Therefore, NfL, as an indicator of neuronal injury, may not be a reliable biomarker for SPSD without the presence of encephalitic symptoms.

Our findings support the use of plasma NfL as a potential minimally-invasive biomarker for disease activity in patients with AINDs with CNS involvement. Our study was limited by several factors, most prominent of which are the relatively small sample size, the heterogeneity of the AINDs studied, the confounder of age between groups studied, and the irregular intervals of longitudinal sampling. Given the small sample size, it may be that our analysis was weighted toward particular AE or AIND subtypes or that it was weighted due to greater prevalence of a particular confounder. If this was the case, our sample may not be indicative of the AE or AIND population as a whole, limiting the generalizability of our individual findings.

The breadth of the AINDs studied represents a trade-off between sample size and homogeneity of the AINDs. For our study, we chose a broader inclusion of AINDs, and worked to show characteristics of subtypes of AINDs, analyzing these subtypes individually as able. However, for the analysis of the correlation of CSF and plasma NfL levels and the longitudinal response of plasma NfL to treatment, we included all AINDs to provide appropriate level of detail in analysis. It may be that these relationships hold for only certain AIND subtypes–such as AE or autoimmune cerebellar ataxias, or for NSAS alone, or for individual autoantibody syndromes. Studies including a larger population of each AIND or individual autoantibody syndrome may be able to better define and confirm these relationships as being specific for an AIND subtype or generalizable.

Age and NfL levels have a known correlation–NfL levels increase as age increases. While we were not able to completely age and gender-match our AIND population to healthy controls, we did have a non-inflammatory neurological disease control population in patients diagnosed with primary headache syndromes, in addition to a small chronic AE/CA cohort with similar age demographics to our active AE cohort. One limitation in this study, however, is that the non-inflammatory headache control population was a younger age group (median 48 yo, range 27–63 yo) when comparing to the AE/CA study population. It is notable that all patients with active AE had plasma NfL levels higher than those found in all age groups (range 20–68 yo) in a study measuring serum NfL levels in 79 healthy individuals (27).

Another limitation of this study is the retrospective, cross-sectional design. For our non-active AE/CA patient group, we did not have longitudinal samples for each patient to evaluate the trend of plasma NfL levels over time. For the three patients with plasma NfL levels above our control range, patients were 7, 8, and 9 months out from their last new or worsening of symptoms and the ages of each patient were 46, 78, and 73 years old respectively. For the 46-year-old-female with non-active GABA-aR AE, her NfL level continued to trend downward at 12 months. For the other two patients at 8 and 9 months, it is unclear if they would have continued to have this downward trend in their NfL levels or if these higher levels represent an underlying chronic neurodegeneration, particularly at the ages of 78 and 73 years old. Additionally, chronically high levels of NfL could represent a prognostic biomarker for persistent neurobehavioral symptoms perhaps related to a chronic neurodegenerative process following AE or CA. In a study measuring exosomal and plasma levels of NfL in mild TBI, those injures associated with higher NfL levels, even years after injury, the greatest elevation were seen in those patients with ongoing neurobehavioral symptoms including postconcussive syndrome, posttraumatic stress disorder and depression (28).

Follow-up sampling for each of our patients was conducted during in-person standard of care visit clinic visits when available–this resulted in irregular sampling intervals for the longitudinal analysis of NfL. To best define NfL as a therapeutic biomarker, regular sampling must be performed to understand the trend of NfL under states of recovery, relapse, and progression and compare between various immunotherapy strategies. For these reasons, larger prospective studies, ideally with standardized intervals of sampling, are needed to understand the longitudinal relationship between NfL and the clinical features, disease severity and long-term outcomes of specific AE and other AINDs.

## Conclusions

Our findings support the potential use of plasma NfL as a minimally-invasive diagnostic and therapeutic biomarker for AINDs with CNS involvement. Further larger, prospective studies are warranted to evaluate the use of NfL in AE and AINDs with the potential to influence decision-making regarding the selection and escalation of immunotherapy and to inform the monitoring and recovery of patients with AE and AINDs.

## Data Availability Statement

The raw data supporting the conclusions of this article will be made available by the authors, without undue reservation.

## Ethics Statement

The study was approved by the Institution Review Board of the University of Colorado, Aurora, CO. The patients/participants provided their written informed consent to participate in this study.

## Author Contributions

All authors contributed to the design and implementation of the research and to the analysis of the results. SS, RK, CM, and AP provided the statistical analysis. CM contributed to sample collection and preparation. CM and AR carried out biomarker and sample testing. AP and RK wrote the manuscript. All authors contributed to review and revisions of the manuscript.

## Funding

This study was supported by the Rocky Mountain MS Center, the Drake family in the name of Susan Drake and grant funding from the Department of Neurology at the University of Colorado.

## Conflict of Interest

RK has received compensation for advisory boards and consultancy with Genentech/Roche. EA has received compensation for activities such as advisory boards, lectures and consultancy with the following companies and organizations: Actelion/Janssen, Alexion, Bayer, Biogen, Celgene/BMS, EMD Serono/Merck, Genentech/Roche, Genzyme. EA has received research support from the following: Biogen, Genentech/Roche, Novartis, TG Therapeutics, Patient-Centered Outcomes Research Initiative, National Multiple Sclerosis Society, National Institutes of Health, and Rocky Mountain MS Center. KN has received grant funding from Genentech and Novartis and consulting fees from Novartis, Biogen, and Bristol Meyers Squibb. TV has received compensation for lectures and consultancy from Biogen, Genentech/Roche, Siranax, Celgene, EMD Serono and Novartis and has received research support from Rocky Mountain Multiple Sclerosis Center, Celgene, Biogen, Anokion, Genentech, F. Hoffmann-La Roche Ltd, GW Pharma and TG Therapeutics, Inc. JB reports personal fees from Roche, personal fees from Genentech, personal fees from Viela Bio, personal fees from Chugai Pharma, personal fees from Alexion, grants and personal fees from Novartis, personal fees from Genzyme, personal fees from Clene Nanoscience, personal fees from Mitsubishi-Tanabe, personal fees from Reistone Bio, grants from National Institutes of Health, outside the submitted work. JB has a patent Aquaporumab issued. AP has received research funding from the Drake Family, Rocky Mountain MS Center, and the University of Colorado through the intradepartmental grant. Outside of this work, AP reports honorarium from MedLink and consulting fees from Genentech/Roche and Alexion. The remaining authors declare that the research was conducted in the absence of any commercial or financial relationships that could be construed as a potential conflict of interest.

## Publisher's Note

All claims expressed in this article are solely those of the authors and do not necessarily represent those of their affiliated organizations, or those of the publisher, the editors and the reviewers. Any product that may be evaluated in this article, or claim that may be made by its manufacturer, is not guaranteed or endorsed by the publisher.

## Abbreviations

AE: Autoimmune Encephalitis
AMPAR: Alpha-amino-3-hydroxy-5-methyl-4-isoxazolepropionic acid receptor
AE: Autoimmune encephalitis
AINDs: Autoimmune neurological disorders
Caspr2: Contactin-associated protein-like 2
CBA: Cell-based assay
CNS: Central nervous system
CA: Cerebellar Ataxia
CSF: Cerebrospinal fluid
DPPX: Dipeptidyl-peptidase-like protein-6
EEG: Electroencephalogram
ELISA: Enzyme-linked immunosorbent assay
GM: Geometric mean
GAD: Glutamic acid decarboxylase
GlyR: Glycine receptor
GFAP: Glial fibrillary acidic protein
GABA-aR: g-aminobutyric acid type A receptor
LGI1: Leucine-rich glioma-inactivated-1
MRI: Magnetic resonance imaging
MOG: Myelin oligodendrocyte glycoprotein
MOGAD: Myelin oligodendrocyte glycoprotein antibody disease
NfL: Neurofilament light
NMOSD: Neuromyelitis optica spectrum disorder
NSAS: Neuronal cell surface antibody syndrome
NMDAR: N-methyl-D-aspartate receptor
SPSD: Stiff person spectrum disorder
TRIM46: Tripartite Motif Containing 46.
